# COVID-19 Impact on Modern Virtual Pathology Education: Challenges and Opportunities

**DOI:** 10.30699/IJP.20201.525144.2589

**Published:** 2021-07-06

**Authors:** Seyed Esmaeil Azimi Khatibani, Shima Tabatabai

**Affiliations:** 1 *Department of Pathology, Amir-Al-Momenin Hospital, Faculty of Medicine, Zabol University of Medical Sciences, Zabol, Iran *; 2 *Department of Medical Education, Shahid Beheshti University of Medical Sciences, Tehran, Iran*

**Keywords:** Education, Implications, Pandemic, Pathology, Virtual platform

## Abstract

**Background & Objective:**

Pathology education conventional methods have been disrupted by the Corona-Virus Disease 2019 (COVID-19) outbreak, compelling a re-evaluation of the means of educational interactions from the undergraduate to the postgraduate level. This commentary explores how the COVID-19 outbreak has challenged pathology education.

**Methods:**

We reviewed the current challenges and determined the potential implications of virtual technologies on modern pathology education for the future of pathology competency learning and assessment.

**Results & Conclusion:**

The challenges are partly due to transferring from in-person teaching to a virtual education. Other reasons are shifting away from discipline-based teaching to organ-system based in medical curriculum and additional pressures on pathology faculties, such as increased demand for pathology services, lack of time, and learning resources. Keeping the national standards in pathology education even in the constant disruptions from pandemic outbreaks are current challenges. Pathology expertise will need to use emergent technologies in providing educational material to ensure quality pathology education. However, virtualization of pathology education produces a value of digital pathology and web-based pathology training materials. Medical students could review clinical cases remotely with their supervisors and gain the pathology competencies necessary for clinical practice.

We need new innovative strategies, and we suggested the following steps to take advantage of the current opportunity to meet the challenges: evaluating the available digital training materials for formal pathology education, investing in the virtual infrastructure for competency-based pathology education, expanding student/residents exposure to pathology educational cases through virtual platforms; applying digital pathology solutions for virtual pathology education.

## Introduction

Pathology education conventional methods have been disrupted by the COVID-19 outbreak, compelling a re-evaluation of the means of educational interactions from the undergraduate to the postgraduate level (1). The challenges are partly due to transferring from in-person teaching toa virtual education. They are also a result of additional pressures on pathology faculties, such as increased demand for pathology services, lack of time, and learning resources (2, 3). This pandemic has increased the need to determine the essential contributions of pathology to diagnose and treat disease and the ongoing pathologists’ tremendous works.

Worldwide, the health systems confront with a shortage of pathologists (4).

Pathology Education in Modern Medical Curricula

Pathology is the vital component of diagnostic strategies and central to medical education (3, 5). Pathology education is conducted based on core principles, including chemistry, biochemistry, anatomy, physiology cell, and genetic theory.

The most medical curriculum has already reformed toward horizontal integration of courses, case presentation in the preclinical phase. The undergraduate medical education shifted away from discipline-based teaching to organ-system based in the medical curriculum. These changes affect pathology education at the undergraduate level. Moreover, forming medical educator teams composed of various medical specialists increasing the role that interdisciplinary teams play in medical education. Integration of disciplines has eliminated formal pathology classes in many medical schools (3, 6). However, this integration has not reduced the importance of pathology in the medical curriculum. Medical students must master these core principles to understand the pathology of organ systems and competency development. 

With current educational policies to reduce transmission of COVID-19, Pathology departments and multidisciplinary networks should evaluate where the virtual educational technologies fit into their new educational plans in an integrated medical curriculum.

Keeping the Competency-based Pathology Education 

The pathology competencies represent the mini-mum requirements of what the national pathology education board has agreed upon to educate undergraduate medical students for practice and for entry into any residency program.

Pathology competency-based education focuses on applying pathology knowledge into practice, clinical reasoning, and pathology diagnostic skills.

Keeping the competency-based standards in pathology education even in constant disruptions from pandemic outbreaks are current challenges of pathologists. 

The ability to gain a broad knowledge of the physiological and pathological processes of each organ system, the competency to apply disease mechanisms to explore the pathobiology, and the competency to improve the pathology diagnostic and therapeutic skills through lifelong learning is essential in becoming a competent physician (7).

Pathology Education Challenges in Shifting to Virtual Learning 

Pathology education's main general challenges are deciding how much to change the courses, adapting to online education, determining the efficiency and effectiveness of virtual learning, and maintaining student engagement (1). The other specific challenges for path-ology discipline are practical teaching and assessment (laboratories, clinical competencies, fieldwork).

We Need new Innovative Strategies, and We Suggested the Following Steps to Take Advantage of the Current Opportunity to Meet the Challenges. However, contextual priorities may lead medical education experts to make individualized decisions based on unique circumstances.

1) **Evaluating the free available web-based pathology training materials for formal pathology education**

The studies indicate that medical students adopted the online resources as a learning tool, and a combina-tion of supervised small group discussions and the self-directed web-based (online) format improved path-ology teaching (8, 9). Web-based pathology educational resources progressively presented in support of both general and system-based pathology teaching. Rich web resources are including a wide variety of visual/audio/text meeting the needs of different learning styles in students.

The educational impact-not availability- of pathology web resources is a key for a successful transition to effective virtual pathology education. Therefore, pathology educational experts should evaluate the online videos according to national standards in pathology education whether they contribute to pathology education at the undergraduate and postgraduate levels. 

Some publicly available websites illustrate pathological principles, present rare disorders that are less relevant to undergraduate education. Some studies indicate that YouTube as a popular online video sharing website is an informative and accurate media of histopathology (cellular pathology) learning for both undergraduates and postgraduates. Moreover, autopsies were present on the site (10).

Besides, Case X is an online platform that provides case-based learning materials and video-based medical cases. Online Case X helps student’s clinical reasoning through self-directed learning (11). Self-directed web-based pathology education provides a rich context for academic pathologists to produce a re-usable form of high-quality pathology cases, enables controlling the quality of macroscopic and microscopic images, utilizing color and dynamic representations, assessing student progress, and providing web-based formative support and access expert feedback.

2) **Investing in the Virtual Infrastructure and Products for Competency-based Pathology Education and Assessment**

The innovative learning technologies will change the future of pathology education and can facilitate training pathology with interactive simulation learn-ing (12). The interactive educational cases should pre-sent in a way to develop clinical reasoning in patholo-gists. In the virtual platform, the pathology competen-cies and learning objectives should be determined clearly for each designed case, clinical presentation, and educational points. 

The virtual educational products need massive infrastructures, including management software and hardware for clinical simulation, case designing tools, and cloud-based eLearning. Pathology content experts should support the virtual system for developing specific learning objectives, pathologic cases, providing updated educational material, virtual supervision, and support (1).

The virtual reality (VR) platform and interactive virtual learning tools can respond to specific issues for pathology discipline that is practical in training and competency assessment (laboratories, clinical skills, fieldwork). The Advancements in VR technology make its use as a training and assessment tool in online objective structured clinical examination (OSCEs) and Virtual OSCEs (VOSCEs) possible (1, 13). 

The formative ‘‘online OSCE’’ for pathology education could assess the disease recognition skills in pathology residents. A sequence of stations presents high-quality specimen images, and the residents should answer the questions and determine the organ and diagnosis within a time limit. After the deadline, they can find the correct answers and case histories of the specimens (9).

Moreover, this can include standardized virtual patient resembling a real patient encounter in virtual stations. Variety of tools are applied in the health care system such as medical history taking, examination, diagnosis, laboratory/ imaging tests, and treating the VP. The system helps medical students master main pathology competencies, including disease mechanisms, organ system pathology, and diagnostic and therapeutic pathology skills. Besides, automated scoring is complementary to assess their progress. Virtual simulation provides the necessary tools to provide benchmarking, better preparing them for real-world medical practice.

3) **Expanding Pathology residents’ Exposure to Educational Cases Through Virtual Platforms**

Previous studies showed that some pathology educational programs should increase the residents’ exposure to pathology cases and practice because pathology subspecialty training programs require residents to apply for fellowships as early as the end of their specialty training. Also, studies indicate that changes in pathology specialty curricula and educa-tional activities have affected resident choices of fellowships (14, 15).

Keeping the national standards in pathology education are current challenges. Pathology expertise will need to use emergent technologies in providing educational material to ensure quality pathology education**. **

The clinical pathology cases presented through a patient’s laboratory data or images by detailed explanations help the learner to understand and apply diagnostic principles and incorporate morphologic or laboratory results for accurate diagnosis and treatment. Virtual discussion forums provide the context for sharing the core educational points to promote clinical reasoning.

4) **Applying Digital Pathology Solutions for Virtual Pathology Education**

Virtualization of pathology education produces a value of digital pathology; it also assists medical universities to support the continuing education of pathologists in the COVID-19 crisis.

Digital pathology (DP) becomes the necessary components in clinical pathology service and cellular pathology training. Pathologists require innovative DP tools and artificial intelligence for digital-enabled care. Residents need these tools to acquire their pathology competencies necessary for the practice of pathology during their early years and discover which subspecialty areas most interest them (15).

DP provides real-time access to clinical/ pathology cases and virtual simulation-based education technologies provide the necessary tools to review clinical cases remotely with supervisors and discuss cases with a histopathologists consultant through virtual conferencing during this pandemic.

Some DP technology products are also ready for deployment on the virtual medical education platforms. 

England’s national health systems (NHS) partners from Oxford, Belfast, Nottingham Universities, and PathLAKE Features University are creating fully digital cellular pathology laboratories with a database of anonymous scanned slide images. These university diagnostic labs are developing AI algorithms for their generated slides (16, 17).

Pathology education is witnessing a paradigm shift where scientific progress and technological advances are growing faster than pathologists’ workforces. Digital pathology and AI algorithm for image analysis would promote pathologic diagnosis. Such technologies and innovations are still in their early stages, but the future is promising. Applying digital pathology solutions for virtual pathology education will increase the efficiency of individual pathologists.

## Conclusion

We have described the challenges of keeping pathology education on track during the pandemic, and how virtual education platforms and digital technologies in pathology can provide real-time solutions to pathology competencies development. Pathology aca-demic departments should evaluate new technologies and encourage routine adoption of virtual education for digital native medical students. 

Moreover, the virtual platforms allowed the pathology educators to use DP with providing a safer and more efficient education during the COVID-19 crisis.

The following four steps were suggested for utilizing the formal pathology education (from the undergraduate to the postgraduate) to support uninterrupted learning and assessment: 

1) Evaluating the free available web-based patho-logy training materials for formal pathology education. 

2) Investing in the virtual infrastructure and pro-ducts for competency-based pathology education and assessment.

3) Expanding pathology residents’ exposure to educational cases through virtual platforms. 

4) Applying digital pathology solutions for virtual pathology education.

The Advancements in DP make it a pathology competencies training and assessment tool. The technologies still need to progress before virtual OSCEs become a comprehensive assessment plat-form. Despite this, the future is promising.
